# Benefits and Limitations of Computer Gesture Therapy for the Rehabilitation of Severe Aphasia

**DOI:** 10.3389/fnhum.2016.00595

**Published:** 2016-11-29

**Authors:** Abi Roper, Jane Marshall, Stephanie Wilson

**Affiliations:** ^1^Division of Language and Communication Science, City, University of LondonLondon, UK; ^2^Centre for Human-Computer Interaction Design, City, University of LondonLondon, UK

**Keywords:** aphasia, speech and language therapy, gesture therapy, computer rehabilitation, communication deficits

## Abstract

Aphasia intervention has made increasing use of technology in recent years. The evidence base, which is largely limited to the investigation of spoken language outcomes, indicates positive treatment effects for people with mild to moderate levels of aphasia. Outcomes for those with severe aphasia, however, are less well documented and – where reported – present less consistent gains for measures of spoken output. This study investigates the effects of a purpose-built gesture therapy technology for people with severe aphasia: GeST+. Study outcomes show significant improvement in gesture production abilities for adults with severe aphasia following computer intervention. They indicate no transfer of effects into naming gains or interactive gesture. Outcomes offer encouraging results for computer therapy methods within this hitherto under-researched population but indicate a need for further refinement of interventions in order to maximize persistence of effects and generalization into everyday communication.

## Introduction

About a quarter of stroke survivors have long-term speech and language difficulties caused by aphasia (Ali et al., 2015). When problems are severe, both speech and writing may be eliminated with profound consequences for the person’s quality of life (Hilari and Byng, 2009). Rehabilitation outcomes in those with severe aphasia are also poor (Plowman et al., 2012).

Although aphasia therapy is making increasing use of technology (van de Sandt-Koenderman, 2011; Zheng et al., 2016), few applications have been designed for people with severe impairments (van de Sandt-Koenderman et al., 2007 for an exception). There is also evidence that some language remediation tools do not benefit this group (Palmer et al., 2012). This study evaluated a bespoke computer therapy for people with severe aphasia targeting the compensatory modality of gesture.

The use of gesture can augment communication in severe aphasia (Goodwin, 2000; Parr, 2007) and has been shown to respond to therapy (Daumuller and Goldenberg, 2010; Marshall et al., 2012). However, gains from gesture therapy are often confined to practiced items, with no generalization beyond these; and, in many studies, it is not clear whether acquired gestures benefit interactive communication (Rose et al., 2013). Outcomes also vary across individuals. This may reflect the severity of the aphasia, or impairments in skills related to gesture production, such as executive function (Purdy and Koch, 2006) and praxis (Hogrefe et al., 2012).

Gestures may not simply replace speech in aphasia, they may also cue speech production (Lanyon and Rose, 2009). This is in line with the theoretical proposal that gestures play a facilitatory role, even in healthy speech production (Krauss et al., 2000). Indeed, treatments that include a gestural component have improved word retrieval in aphasia (Raymer et al., 2006; Crosson et al., 2007; Attard et al., 2013). However, the independent contribution of gesture to the treatment effect is difficult to determine. When gesture is treated in isolation effects have not generalized to speech (Marshall et al., 2012).

Therapeutic gains in gesture production are hard won, particularly when the aphasia is severe. For example previous studies have found that at least 3 h of therapy were needed to train each new gesture (Daumuller and Goldenberg, 2010; Marshall et al., 2012). These findings call for intensive treatment regimes. Yet, such regimes are not always available, and may be associated with high rates of drop out (Brady et al., 2016).

Self-administered computer therapies can raise therapy intensity without increasing therapist demand, and in ways that may inhibit drop out. GeST (Galliers et al., 2012) is a therapy tool designed with and for people with severe aphasia in order to train a ‘vocabulary’ of everyday communicative gestures. It employs computer vision-based gesture recognition to determine whether the user has produced the correct gesture. A number of motivating features promote engagement, including opportunities for different levels of practice, applause, and a ‘gaming’ element involving a 3D virtual world.

A pilot study involving nine people with severe aphasia showed that 6 weeks practice with GeST improved gesture production (Marshall et al., 2013). However, gains were modest and only occurred on items that had been practiced with the tool and with therapist support. Spoken naming of both trained and untrained items was explored, with no evidence of a therapy effect. Use of the acquired gestures in communication was not tested.

This paper reports a new therapy study involving GeST+. It aimed to replicate the positive findings of the pilot, with a larger sample and a stronger, quasi-randomized controlled design. The original GeST tool was augmented (GeST+) with an additional software application, to determine if this would enhance the therapy effect. A wider range of outcome measures, and longer follow up, aimed to identify the potential benefits of GeST+, including for interactive communication. Benefits for speech were also examined, through pre- and post-therapy tests of word production. Finally, we examined whether baseline tests of language, cognition, and praxis predicted therapy outcomes.

## Materials and Methods

This study employed a wait-list control, quasi-randomized design. Screening and profiling assessments were administered at recruitment. Following screening, participants were allocated to either an immediate or delayed therapy group. Allocation was performed by a member of the team (SW) who was blind to screening data. This was achieved via selection of a paper label displaying either **‘immediate’** or **‘delayed’** from an opaque bag which contained a batch of 10 labels – five stating immediate and five stating delayed. Participants were allocated to their group as indicated by the label selected. The first 11 participants to enter the study were recruited in the South East of England. Allocation here was carried out as each participant entered the study. A second block of data collection was carried out in the South West of England. For logistical reasons allocation here was carried out in blocks of five cases at a time, i.e., five participants at a time were allocated to either the immediate or delayed group. This enabled data collection and therapy delivery to be carried out contemporaneously for this group of participants – to overcome logistical limitations imposed by travel requirements.

Assessments were administered at four time points (T1, T2, T3, T4) – each separated by an interval of 5 weeks. Between T1 and T2, those in the immediate therapy group received 5 weeks of computer-delivered gesture therapy, supported by weekly therapist input. Participants in the delayed therapy group received no input. Both groups undertook repeated measures testing at T2. Following this, those in the delayed group received the 5-week therapy protocol, whilst those in the immediate group received no input. Repeated measures’ testing was carried out again at T3 for both groups. T4 testing was carried out after a further 5 weeks of no input for either group. Computer log data captured therapy practice time and system usage information.

Repeated measure testing at all time points included three main assessments: a primary assessment of gesture production in isolation and secondary assessments of gesture in interactive communication and spoken naming.

### Screening and Profiling Assessments

A number of assessments were included to screen and profile participants’ abilities in language, cognition, and praxis. These were included to investigate links between such abilities and any subsequent gains made on the primary or secondary outcome measures.

#### Language

Four subtests from the standardized Comprehensive Aphasia Test [CAT, (Swinburn et al., 2004)] were employed to assess language. The CAT single word spoken naming assessment was used as a screening measure, with participants scoring 30% or under (i.e., with severe naming difficulties) being included in the study. Following screening, three further CAT subtests were used to profile participants’ individual language abilities: *spoken single word comprehension, sentence comprehension*, and *written single word comprehension*.

#### Cognition

These tests included a basic object to picture matching assessment and a standardized measure of non-verbal cognition.

##### Object and gesture to picture matching

This screening assessment examined participants’ ability to relate objects and gestures to both photographs and line drawings – skills which were required for completion of the experimental measures, and for successful use of GeST. It was based on a novel assessment describe in Caute et al. (2013) and used to screen participants in two previous gesture studies (Marshall et al., 2012, 2013). Participants scoring 60% or less (≤6/10) for this assessment were excluded from progression to the main study.

##### Visuospatial assessments from the Cognitive Linguistic Quick Test

Following Nicholas et al. (2011) in assessment of cognition for adults with severe aphasia, visuospatial skill domain subtests from the Cognitive Linguistic Quick Test (CLQT; Helm-Estabrooks, 2001) were employed as profiling tests to assess participants’ non-linguistic cognition. These comprised standardized assessments of *symbol deletion*; *symbol trails*; *design memory, mazes*, and *design generation*.

#### Praxis

The Birmingham University Praxis Scale (BUPS, as cited in Bickerton et al., 2012 and standardized for use with both chronic and acute stroke patients) was used as a profiling assessment to examine participants’ fine and gross motor skills and core gesture abilities. The measure comprised four subtests – each supplying written prompts alongside spoken instruction and thus reducing linguistic and memory demands for participants. Subtests examined multi-step object use; gesture production; gesture recognition and meaningless gesture imitation as described in Bickerton et al. (2012).

### Primary Gesture Assessment

The primary gesture assessment comprised gesture production from a picture. Derived from a measure employed by Marshall et al. (2013) in the pilot study of GeST, this assessment comprised 40 photograph images of individual objects pictured against a white background. Twenty of the objects presented were items trained in the study. A further 20 were items matched for lexical frequency. Participants were shown an image of an object and instructed: “Show me a gesture for this. Use your hands and your face.”

Participant gesture production was video recorded. Using a method described in Marshall et al. (2012, 2013), videos were later edited into a series of four new videos, each comprising the 40 gestures elicited but distributed across a range of time points. These videos were shown to scorers blinded to the target item, time of assessment and group allocation of the participants. Scorers were asked to identify the item being gestured on a written response sheet. The responses were then compared to the target item and scored for accuracy – with two points being awarded for each accurate identification or acceptable synonym (see Supplementary Data Table 1 for a full list of synonyms), one point for a semantically acceptable alternative (see Supplementary Data 2 for a full description of the scoring process), and 0 points for no response or where the participant indicated that they did not know the answer. A maximum score of 40 points was available for treated items and 40 points for untreated items in this assessment. To evaluate the reliability of the scores awarded for the gesture, videos for 22 of 77 gesture assessments (29%) were viewed and scored by second scorer. Selection of these videos was distributed evenly across the participant data. The second scorer was blinded to the design of the project and the time point at which the assessment had been conducted. The overall percentage of agreement between scorers was 86%.

### Interactive Gesture Assessment

Secondary gesture assessment was assessed using a novel measure developed for the purposes of this study. Assessment comprised live gesture production for a familiar communication partner (a family member, friend, or carer in the participant’s immediate environment recruited at the same time as the participant). Participants were shown a short video clip of an everyday situation (e.g., a person answering a telephone) using a Microsoft PowerPoint presentation. Immediately following the clip, a still photograph of a relevant object from the video (e.g., a telephone) appeared against a white background and the participant was asked to gesture the object. The participant was instructed: “I’m going to show you a short video. At the end of the video is a picture. Your job is to gesture that picture to X (like a game of charades). X will try to work out who or what it is and write it down.” Two practice items were shown, followed by 12 test items. The interactive charades assessment had four alternate versions: A, B, C, and D. Each contained six treated and six untreated test items. The order of presentation was randomly assigned and was different for each version. As for the primary gesture assessment, participants were awarded two points for each accurate identification or acceptable synonym, one point being for a semantically acceptable alternative and 0 points for no response or where the participant indicated that they did not know the answer. A maximum score of 12 points was available for treated items and 12 points for untreated items in this assessment.

### Naming Assessment

Naming assessment comprised spoken picture naming. Items employed were identical to those used in the primary gesture assessment (see Screening and Profiling Assessments). Participants were presented with a photograph image of an object and asked to state the name of that object. Responses were transcribed/recorded by the researcher and scored for accuracy. A maximum score of 40 points was available for this assessment. Assessment was video recorded for subsequent inter-rater reliability measures. To evaluate the reliability of the scores awarded for the naming data, videos for 14 of 77 naming assessments (18%) were viewed and scored by a researcher external to the project. These videos had been randomly selected using a computer-based randomization process. The second scorer was blinded to the design of the project and the time point at which the assessment had been conducted. A two-way, mixed method intraclass correlation (ICC) was conducted to compare outcomes from the second scorer to those reported by the primary researcher. A high degree of reliability was found between the two score sets. The average measure ICC was 0.907 with a 95% confidence interval from 0.657 to 0.972 [*F*(13,13) = 27.81, *p* < 0.001].

### Therapy Protocol

Therapy was delivered to participants in their homes over a period of 5 weeks. Weeks one to four adhered to the protocol described by Marshall et al. (2013) in the GeST pilot study. Briefly, participants were presented, one at a time, with gesture videos within GeST and instructed to repeat them. Accuracy was monitored using vision-based gesture recognition and applause was supplied for each correctly produced gesture. Each week a speech and language therapist completed a familiarization exercise with the participant – introducing them to the five gestures to be practiced that week. This was followed by around up to an hour’s supported practice with the computer. Participants were then asked to practice independently for around an hour each day. Week five introduced a supplementary consolidation exercise, allowing participants to practice all 20 gestures together. This cumulative practice period aimed to address the shortcoming noted in (Marshall et al., 2013) that limitations in therapy gains may arise as a result of practicing successive gesture batches instead of a full set. Video clips of the individual gestures were presented, one at a time, using a PowerPoint show. Participants were instructed to copy the gesture demonstrated in the clip. We use the term GeST+ to refer to the combined application of the GeST tool and the supplementary consolidation exercise. Following completion of the 5 weeks of therapy, participants received no further access to GeST+. Additionally, those in the delayed treatment group received no access to GeST+ outside of the allotted treatment period.

### Ethical Approval

Ethical approval for this study was granted by the City, University London School of Community and Health Sciences Research Ethics Committee. Following the provision of accessible written and verbal information, all participants gave written informed consent in accordance with the Declaration of Helsinki.

### Hypotheses

For each outcome measure (primary or secondary), we predicted an improvement in performance following intervention and a maintenance of this effect after a further five and (for the case of the immediately treated group) 10 weeks. It was anticipated that this effect would be greater for items treated within the intervention protocol when compared to those that were untreated. In addition, a relationship between the screening/profiling assessment scores and changes in outcome measure performance was predicted, as was a relationship between levels of GeST+ practice and changes in outcome measures.

### Data Analysis

Primary and secondary outcome measures were subject to two ANOVA analyses. Unless otherwise stated, data met ANOVA assumptions. When this was not the case, log transformations were applied. The first analysis was a mixed within and between subject ANOVA conducted on data collected at T1 and T2. The within variables were time and item. The latter contrasted items that had been treated in GeST+ with items that had not been treated. The between variable was group: immediate vs. delayed. Participants in the immediate treatment group had received GeST+ therapy between T1 and T2, whereas participants in the delayed treatment group had not. Thus a treatment effect was signaled by a time by group interaction. Time by item interactions indicated whether treatment effects were specific to items practiced in GeST+.

The second analysis was a within group ANOVA conducted on the pooled data across all participants, i.e., the immediate and delayed groups combined. The variables were time and item. Time had three levels: pre-therapy (conflating T1 for immediate and T2 for delayed), post-therapy (conflating T2 for immediate and T3 for delayed), and 5 week maintenance (conflating T3 for immediate and T4 for delayed). Item had two levels: treated and untreated. Here treatment effects were indicated by a significant main effect of time, with significant planned comparisons between pre- and post-therapy. Significant comparisons between pre-therapy and maintenance suggested that changes were still evident 5 weeks post-therapy. Time by item interactions again indicated whether treatment effects were specific to items practiced in GeST.

Participants in the immediate treated group underwent a second maintenance assessment, 10 weeks after the end of therapy (T4). Longer-term maintenance of change in this group was assessed by paired *t*-test comparisons comparing scores at T1 and T4.

Finally, correlation analyses aimed to determine whether any of the screening or profiling assessments were predictive of gains on the outcome measures. Gains were determined by subtracting the pre-therapy from the post-therapy test scores (T2–T1 for immediate; T3–T2 for delayed). GeST usage times were also correlated with gain scores to explore the influence of dose.

## Results

### Participants

**FIGURE 1:**
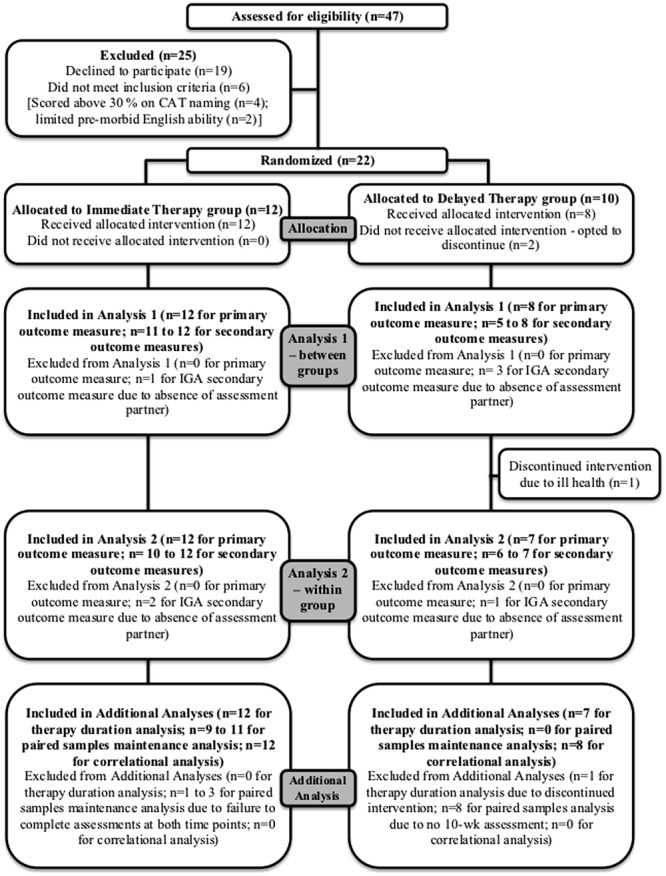
**CONSORT diagram showing participants’ progression through the study**.

Twenty-two participants were recruited. Following randomization, two opted to discontinue. Twenty participants were therefore included in the analysis. **Figure 1** shows participants’ progression through the study. All participants had experienced a left hemisphere stroke with resultant severe aphasia and hemiplegia. Only one retained use of his right hand.

**Table 1** reports the demographic and screening/profiling test scores for participants in the immediate (*n* = 12) and delayed (*n* = 8) intervention groups. *T*-test and Mann–Whitney comparisons confirmed that there were no significant differences between the groups with respect to age, time post-stroke, or any of the test scores. All bar three participants had some experience of computers. The delayed group reported slightly higher pre-stroke computer use.

**Table 1:** Participant information.

	Group allocation	Participant numbers within each category
Gender	Immediate	7 male; 5 female
	Delayed	6 male; 2 female
Pre-stroke reported computer use	Immediate	3 never; 4 once a week or less; 5 almost every day
	Delayed	0 never; 2 once a week or less; 6 almost every day

	**Immediate group mean (*SD*)**	**Delayed group mean (*SD*)**

Age (in years)	67.83 (10.18)	67.00 (10.71)
Total number of months post-stroke (at T1)	61.42 (71.18)	54.86 (38.58)
CLQT visuospatial cognitive domain score (/105)	45.67 (24.87)	48.13 (26.90)
BUPS non-standardized praxis summary (/42)	21.25 (9.67)	18.88 (9.05)
CAT picture naming subtest raw score (/24)	0.42 (1.16)	0.88 (2.10)
CAT spoken Word comprehension subtest raw score (/15)	11.17 (2.72)	11.75 (2.82)
CAT spoken sentence comprehension subtest raw score (/16)	6.17 (2.59)	6.63 (1.69)
CAT written word comprehension subtest raw score (/15)	9.08 (3.94)	8.88 (3.18)

### Usage

The time spent using GeST was automatically logged. Across all participants (immediate and delayed groups combined) the mean usage time was 14 h, 50 min (range 5:20–26:50). The mean number of sessions was 52.05 (range 22–132). Usage was highest in the first week, with a mean of just over 5 h. After this, mean usage was close to 3 h per week.

### Analysis 1: Mixed within and between Subject ANOVAs

T1 and T2 scores on the primary and secondary outcome measures are reported in **Table 2**. There were missing data on the Interactive Gesture Assessment (IGA) owing to partners being unavailable.

**Table 2:** Mean scores (SD) on the outcome measures at T1 and T2 for the immediate and delayed groups.

Assessment		Group	T1 score (*SD*)	T2 score (*SD*)
Gesture assessment	Treated items (max. score = 40)	Immediate (*n* = 12)	6.75 (5.86)	11.33 (6.80)
		Delayed (*n* = 8)	9.38 (6.59)	9.13 (5.46)
	Untreated items (max. score = 40)	Immediate (*n* = 12)	5.00 (4.07)	5.75 (4.83)
		Delayed (*n* = 8)	5.88 (4.29)	4.88 (5.41)
Interactive gesture assessment	Treated items (max. score = 12)	Immediate (*n* = 11)	6.01 (3.36)	6.27 (3.66)
		Delayed (*n* = 5)	4.60 (2.97)	6.66 (3.13)
	Untreated items (max. score = 12)	Immediate (*n* = 11)	5.10 (3.08)	4.73 (4.43)
		Delayed (*n* = 5)	4.40 (3.78)	6.20 (3.56)
Naming assessment	Treated items (max. score = 40)	Immediate (*n* = 12)	1.83 (2.79)	2.75 (3.47)
		Delayed (*n* = 8)	1.13 (0.99)	0.63 (0.74)
	Untreated items (max. score = 40)	Immediate (*n* = 12)	0.83 (1.53)	0.75 (1.36)
		Delayed (*n* = 8)	0.63 (1.06)	1.00 (1.93)

#### Primary Outcome Measure

##### Gesture assessment

Outcomes from the Shapiro–Wilk test indicated that T1 scores were not normally distributed. Log transformations were therefore applied to all scores. The transformed data met ANOVA assumptions, so were used in the analysis.

The mixed ANOVA revealed a main effect of item [*F* = 39.29 (1,18), *p* < 0.05; η_p_^2^= 0.69], but no effect of time [*F* = 1.97 (1,18), *p* > 0.05, η_p_^2^ = 0.10] or group [*F* = 0.06 (1,18), *p* > 0.05, η_p_^2^ = 0.00]. The item effect indicated that treated items were gestured more successfully than untreated items. There was a significant interaction between group and time [*F* = 10.88 (1,18), *p* < 0.005; η_p_^2^ = 0.38] and between time and item [*f* = 7.77 (1,18), *P* < 0.05; η_p_^2^ = 0.30]. The former signals a treatment effect. The immediate group, who had received intervention between T1 and T2 improved; whereas the as yet untreated delayed group did not. The time by item interaction indicates that treatment gains were largely confined to items that were practiced in GeST+. The three-way interaction was not significant.

#### Secondary Outcome Measures

##### Interactive Gesture Assessment

The mixed ANOVA demonstrated no main effect of time (η_p_^2^ = 0.10), item (η_p_^2^ = 0.17), or group (η_p_^2^ = 0.00). There were no significant interactions (group × time η_p_^2^ = 0.12; group × item η_p_^2^= 0.07; time × item η_p_^2^ = 0.01; group × time × item η_p_^2^ = 0.00).

These results suggest that treatment did not change performance on this measure. Indeed, the descriptive statistics indicate that (marginal) gains were more evident for the delayed group.

#### Naming Assessment

T1 and T2 naming scores were not normally distributed, so log transformations were applied.

The mixed ANOVA demonstrated no main effect of time (η_p_^2^ = 0.01), item (η_p_^2^ = 0.23), or group (η_p_^2^ = 0.01). There were also no significant interactions (group × time η_p_^2^ = 0.10; group × item η_p_^2^ = 0.06; time × item η_p_^2^ = 0.01; group × item × time η_p_^2^ = 0.14). Thus no effect of therapy was seen on the naming assessment in this analysis.

### Analysis 2: Within Subject ANOVAs

Pre-therapy, post-therapy, and 5 week maintenance scores on the outcome measures for all participants (immediate and delayed combined) are reported in **Table 3**. One participant in the delayed group discontinued therapy due to ill-health, so *n* = 19. Missing IGA data were due to partner non-availability.

**Table 3:** Mean (SD) pre-therapy, post-therapy, and 5 week maintenance scores on the outcome measures; immediate and delayed groups combined.

Assessment		Pre-therapy score (*SD*)	Post-therapy score (*SD*)	5 week maintenance score (*SD*)
Gesture assessment (*n* = 19)	Treated items (max. score = 40)	7.84 (5.80)	11.32 (6.53)	11.32 (6.96)
	Untreated items (max. score = 40)	5.21 (4.48)	6.00 (5.17)	6.26 (5.56)
Interactive gesture assessment (*n* = 16)	Treated items (max. score = 12)	6.38 (2.96)	7.31 (3.11)	8.50 (3.18)
	Untreated items (max. score = 12)	5.56 (2.94)	4.75 (3.97)	6.06 (3.94)
Naming assessment (*n* = 19)	Treated items (max. score = 40)	1.37 (2.31)	2.52 (3.15)	1.58 (2.34)
	Untreated items (max. score = 40)	0.95 (1.68)	0.68 (1.25)	0.68 (1.20)

#### Primary Outcome Measure

##### Gesture assessment

**FIGURE 2:**
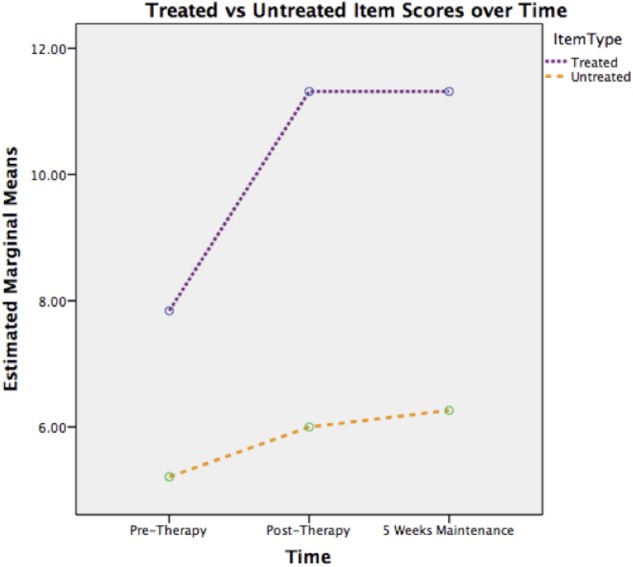
**Treated versus untreated gesture scores over time**.

The within subject ANOVA revealed a main effect of time [*F* = 8.88 (2,36), *p* < 0.005, η_p_^2^ = 0.33] and of item [*F* = 25.02 (2,18), *p* < 0.005, η_p_^2^= 0.58]. The interaction between time and item was not significant [*F* = 2.42 (2,36), *p* > 0.05, η_p_^2^ = 0.12]. Despite the latter finding, the descriptive statistics suggest that gains occurred largely on treated items (**Figure 2**).

Planned comparisons were significant for pre-therapy vs. post-therapy and for pre-therapy vs. maintenance (both *p* < 0.05); but not for post-therapy vs. maintenance. Thus change occurred over the therapy period and was maintained for 5 weeks after therapy was withdrawn.

#### Secondary Outcome Measures

##### Interactive Gesture Assessment

The within subject ANOVA revealed a main effect of time [*F* = 4.31 (2,30), *p* < 0.05, η_p_^2^ = 0.22] and of item [*F* = 90.09 (1,15), *p* < 0.01, η_p_^2^ = 0.57]. The interaction between time and item was not significant [*F* = 2.58 (2,30), *p* > 0.05, η_p_^2^ = 0.22]. The only significant planned comparison was between pre-therapy and maintenance (*p* < 0.05).

**FIGURE 3:**
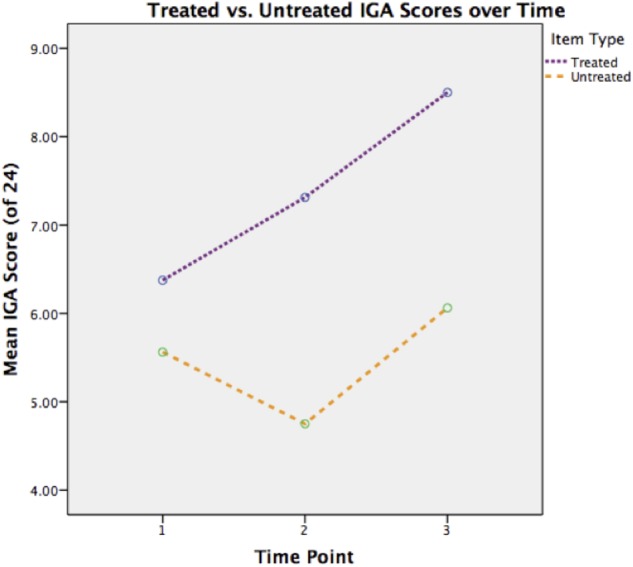
**Treated versus untreated Interactive Gesture Assessment (IGA) scores over time**.

Thus scores on this measure improved over the three testing periods. Although the interaction was not significant, gains were most evident for treated items (**Figure 3**). However, as for the gesture assessment, treated items scored more highly even before therapy began.

#### Naming Assessment

**FIGURE 4:**
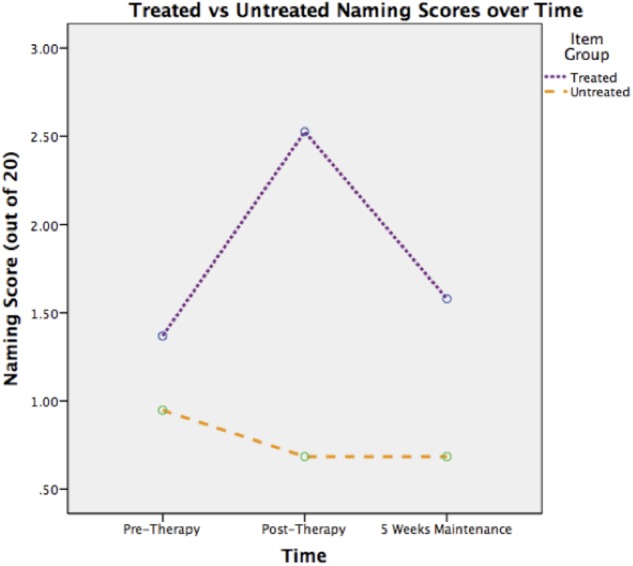
**Treated versus untreated naming scores over time**.

As pre-therapy naming scores were not normally distributed, the mixed ANOVA was performed on log-transformed data. The main effect of time was not significant (η_p_^2^= 0.15). There was a main effect of item [*F* = 7.07 (1,18), *p* < 0.05, η_p_^2^ = 0.28] and a significant interaction between time and item [*F* = 3.63 (2,36), *p* < 0.05, η_p_^2^= 0.17]. **Figure 4** illustrates this interaction. It shows that naming of treated items improved after therapy, but effects were not maintained 5 weeks later.

### Maintenance at 10 Weeks

Eleven participants in the immediate group were available for follow up assessment at 10 weeks post-therapy. Their mean T1 (pre-therapy) and T4 (10 weeks maintenance) scores are reported in **Table 4** and were subject to paired *t*-test comparisons. The only significant result was for treated items in the IGA [*t*(10) = 2.47, *P* < 0.05].

**Table 4:** Mean scores (SD) on the outcome measures for the immediate group at T1 and T4 (*n* = 11).

Assessment		T1 Score (*SD*)	T4 Score (*SD*)
Gesture assessment	Treated items (max. score = 40)	7.36 (5.73)	9.82 (7.39)
	Untreated items (max. score = 40)	5.45 (3.93)	5.91 (4.66)
Interactive gesture assessment	Treated items (max. score = 12)	6.45 (3.08)	8.82 (3.12)
	Untreated items (max. score = 12)	5.73 (3.10)	5.91 (3.73)
Naming assessment	Treated items (max. score = 40)	2.00 (2.86)	2.36 (3.26)
	Untreated items (max. score = 40)	0.91 (1.58)	1.36 (1.86)

### Correlation Analyses

Gain scores on each of the outcome measures (derived by subtracting the pre- from the post-therapy scores) were correlated with screening/profiling test scores and with individual GeST usage times. These correlations aimed to determine whether any of the baseline skills of participants were prognostic of gain, and whether there was an association between dose (GeST practice times) and gain.

None of the correlations was significant and values were low, with only one exceeding 0.4. This was between the Gesture Assessment gain and CAT spoken word comprehension score (*r* = 0.42).

## Discussion

In this study, 20 people with severe aphasia were offered 5 weeks of GeST+ therapy, comprising 4 weeks practice with GeST and a week of consolidation with another software application. It was hypothesized that therapy would improve the production of gestures in isolation (primary outcome measure) and when conveying an item to a conversation partner (secondary outcome measure). The influence of therapy on spoken word production (a further secondary outcome measure) was also explored via a picture naming test. All outcome measures included items that had been treated in therapy and matched, untreated items, with anticipated benefits for the treated items. Additional analyses explored maintenance of gain 10 weeks after therapy ceased, and whether any baseline assessments were predictive of gain. The influence of GeST usage times on outcomes was also assessed.

### Participants

All participants in the study had long-term severe aphasia. The mean time post-stroke was over 5 years, yet none had recovered functional speech and most had persisting comprehension difficulties. Even single word production was minimal, with a mean CAT picture naming score below 1. Other stroke related impairments were also evident. Impaired CLQT scores pointed to visuospatial and/or cognitive problems. Group scores on the BUPS assessment of praxis were also impaired, with seven participants scoring less than 50% on this measure.

### Participant Compliance

Despite the severity of participants’ impairments, compliance with the treatment protocol was good. Only one person did not complete therapy, owing to ill health. Across both groups, participants undertook an average of 14 h of self directed practice in GeST. The sessional data indicated that most people used GeST every day, and some practiced much more frequently. Observations of participants using GeST confirmed that all could navigate the tool to access gesture practice. For example, virtually everyone was observed to switch between therapy levels. It seems, therefore, that GeST was accessible to people with severe aphasia and other stroke related impairments. Problems of drop out, which have occurred in other aphasia therapy studies (Brady et al., 2016) were low.

### Outcomes

Turning to outcomes, the hypothesis that GeST+ would improve gesture production on the primary outcome measure was upheld. Both ANOVA analyses supported this conclusion. In the mixed ANOVA, a significant interaction between time and group showed that scores on the gesture assessment improved between T1 and T2 for the immediately treated group, but not for the delayed group. In the within subject ANOVA, which combined data from both groups, there was a significant main effect of time, with planned comparisons showing that performance improved immediately after therapy with maintenance 5 weeks later. Data for both analyses suggested that gains were largely confined to treated items. In line with this, the time by item interaction was significant in the mixed ANOVA. We can conclude that GeST+ therapy improved the production of gestures in isolation. It is important that this gain occurred on a measure that was scored by independent assessors, who were blinded to time point and group allocation.

Scores on the secondary outcome measure for gesture were less encouraging. This task involved gesturing 12 items (six treated and six untreated) to a conversation partner, and aimed to determine whether GeST+ therapy improved the use of gestures in interactive communication. The first mixed ANOVA showed no therapy effects. The more highly powered within group ANOVA produced a main effect of time, indicating that performance improved. However, only the pre-therapy vs. 5 weeks maintenance comparison was significant. As with the primary outcome measure, the data suggested that gains occurred largely on treated items, although the interaction was not significant. Thus, the hypothesis that GeST+ would improve interactive gesture was not upheld.

The final outcome measure explored the effect of GeST+ therapy on spoken naming. The inclusion of this measure was motivated by the theoretical proposal that gestures may facilitate speech (Krauss et al., 2000), and by previous therapy studies that have successfully employed gestures to cue word retrieval (Raymer et al., 2006; Crosson et al., 2007; Attard et al., 2013). The first, mixed ANOVA analysis of the naming data was not significant. However, the within group ANOVA produced a significant interaction between time and item. This arose because, across all participants, naming of the treated words improved after therapy. The improvement was not maintained at 5 weeks follow up and was narrow (mean gain of one item).

Why did naming improve, albeit by a small and transient margin? This may point to cross modality facilitation, with gestures stimulating speech. However, a *post hoc* analysis pointed to a rather different explanation. The presentation of stimuli in GeST+ often included the spoken name; e.g., participants heard the video instruction say: ‘here is the gesture for (name of item).’ Several participants were observed to repeat the names while they attempted the gestures. The *post hoc* analysis determined how many of the target words were repeated by each participant in the final GeST+ session (this session was filmed), and correlated this with the individuals’ pre- to post-therapy naming gains. The finding was significant (*r* = 0.71; *p* < 0.05). It seemed that some participants used retained repetition skills to incorporate speech practice into therapy; and this may have stimulated the narrow and fleeting post-therapy naming gain.

Additional analyses explored the longer-term maintenance of gain. Those in the immediately treated group underwent two follow up assessments, the second of which occurred 10 weeks after therapy ceased. Disappointingly, most scores at this point were found to be no different from the T1 baseline; and although one comparison was significant, the α value (*p* < 0.05) raises concerns about type one error. A recent Cochrane Review of speech and language therapy post-stroke argued that durable change is often not demonstrated (Brady et al., 2016). This was the case here.

Finally, prognosticators of gain were explored. As in the previous study of GeST therapy (Marshall et al., 2013) individual gain scores on all measures varied. For example, three individuals improved by more than 10 points when gesturing treated items, whereas seven made no improvement. These variations suggested that some people were better candidates for GeST+ therapy than others, and this, in turn may relate to their abilities in language, cognition, or praxis. However, correlations between gain scores and baseline measures of these abilities were all insignificant. There was also no relationship between the amount of practice undertaken with GeST and levels of gain.

### Limitations

Before considering the clinical significance of these findings some study limitations need to be acknowledged. Group allocation was not fully randomized and numbers in each group were low. The lack of power was confounded by missing data, particularly in the IGA. The latter was a novel measure – which may not have been sensitive to change. It was also influenced by partner skills, which were untreated. Tests were not administered by blinded assessors, although the scoring of the primary outcome measure was blind.

## Conclusion And Implications For Future Research

This study replicated our previous finding that people with severe aphasia can use GeST to improve their gesture production (Marshall et al., 2013). In the context of severe and intractable impairments such an improvement is clinically important. As previously, usage logs also showed that the application of technology successfully augmented the treatment dose, since just 5 h of therapist support was supplemented by considerable independent practice. However, gains on the primary outcome measure were modest, despite the fact that GeST was augmented with an additional software application (GeST+). There was also no clear benefit for interactive communication and durability was poor. Further research could usefully explore whether using GeST alongside ‘*in vivo*’ communication activities would enhance gains and promote transfer to interactive communication. Providing long-term access to GeST, after formal therapy has ceased, might also maintain improvements. Perhaps most importantly, we need to determine candidacy, or identify those who are most likely to benefit from GeST. In the current study, baseline tests of language, cognition, and praxis were not informative. Collecting a much larger data set ‘in the wild,’ by releasing GeST to practitioners, may be the best way to inform this question.

## Author Contributions

The study was designed by AR, JM, and SW. AR implemented the design, collected results, and conducted analysis under the supervision of JM and SW. AR and JM wrote the initial working draft of the paper. SW provided critical revision. AR completed writing of the paper and contributed the design and presentation of figures.

## Conflict of Interest Statement

The authors declare that the research was conducted in the absence of any commercial or financial relationships that could be construed as a potential conflict of interest.
