# Supplementary material for: Benefits and Limitations of Computer Gesture Therapy for the Rehabilitation of Severe Aphasia
Source: Front Hum Neurosci. 2016 Nov 29;10:595. doi: 10.3389/fnhum.2016.00595 (PMC5126070; doi:10.3389/fnhum.2016.00595)
Supplement: Supplementary file 1 [file Data_Sheet_1.docx]

Supplementary Material

Benefits and limitations of computer gesture therapy for the rehabilitation of severe aphasia

Abi Roper^*^, Jane Marshall, Stephanie Wilson

*** Correspondence:** Abi Roper: Abi.Roper.1@city.ac.uk

# Supplementary Data 1. List of acceptable synonyms used in scoring

# [Synonym items generated using WorldNet 3.1 (Princeton University, 2010)]

**Supplementary data table 1.**

| Item | Synonyms |
| --- | --- |
| Apple | Apple, orchard apple tree, Malus pumila |
| Banana | Banana |
| Bed | Bed |
| Beer | Beer |
| Book | Book, volume |
| Boy | Male child, boy, son |
| Camera | Camera, photographic camera |
| Car | Car, auto, automobile, motorcar, machine |
| Cat | Cat, true cat |
| Chess | Chess, chess game |
| Cup | Cup |
| Dentist | Dental practitioner, dentist, tooth doctor |
| Dentures | Denture, dental plate, plate |
| Doctor | Doctor, doc, physician, MD, Dr., medico |
| Door | Door |
| Food | Nutrient, food, solid food |
| Football | Football, football game |
| Glasses | Specs, spectacles, eyeglasses, glasses |
| Gloves | Glove |
| Hair | Hair |
| Hat | Hat, chapeau, lid |
| Iron | Iron, smoothing iron |
| Letter | Letter, missive |
| Money | Money |
| Newspaper | Newspaper, paper |
| Pen | Pen |
| Piano | Piano, pianoforte, forte-piano |
| Rain | Rain, rainfall |
| Rainbow | Rainbow |
| Remote Control | Remote, remote control |
| Scissors | Scissors, pair of scissors |
| Sewing | Sewing, stitching, stitchery |
| Spider | Spider |
| Sponge | Sponge |
| Stamp | Stamp, postage, postage stamp |
| Swimming | Swimming, swim |
| Tap | Water faucet, water tap, tap, hydrant |
| Tea | Tea |
| Telephone | Telephone, telephone set, phone |
| Tissue | Tissue, tissue paper |
| Umbrella | Umbrella |
| Waiter | Waiter, server |
| Walking Stick | Walking stick |
| Watch | Watch, ticker |
| Wife | Wife, married woman |
| Wine | Wine, vino |

**
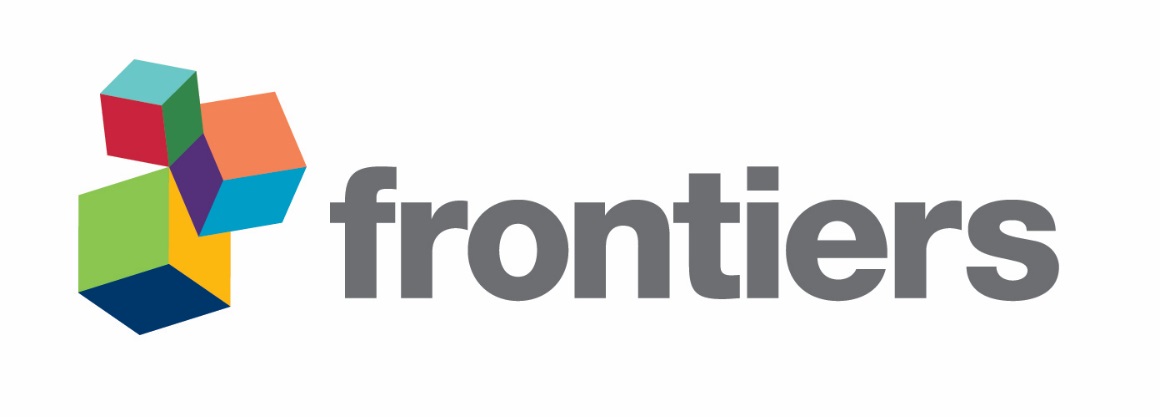
**
