# Supplementary material for: Benefits and Limitations of Computer Gesture Therapy for the Rehabilitation of Severe Aphasia
Source: Front Hum Neurosci. 2016 Nov 29;10:595. doi: 10.3389/fnhum.2016.00595 (PMC5126070; doi:10.3389/fnhum.2016.00595)
Supplement: Supplementary file 2 [file Data_Sheet_2.docx]

Supplementary Material

Benefits and limitations of computer gesture therapy for the rehabilitation of severe aphasia

Abi Roper^*^, Jane Marshall, Stephanie Wilson

*** Correspondence:** Abi Roper: Abi.Roper.1@city.ac.uk

# Supplementary Data 2

# Scoring Criteria for gesture assessments

Those responses that matched the target item or a synonym of the target item exactly were awarded 2 points. [Synonym items were generated using WorldNet 3.1 (Princeton University, 2010). A full list of synonyms is provided in data sheet 1.] Those responses where a scorer or participant had supplied no response or indicated that they did not know the answer, were awarded 0 points. All other responses were deemed ambiguous and were subject to a further scoring judgment by a second scorer to examine their semantic acceptability. This process was conducted as follows for each target word – ambiguous-response pair:

1. A list of the target words was created (list 1). This list was duplicated to give a list double in size and containing each of the target words twice.
2. A list of the ambiguous responses to those targets was created (list 2). This list was duplicated to give a list double in size and containing each of the ambiguous response words twice.

Responses from list 2 were linked once to their intended target and once to a randomly selected word from list 1 (i.e. a foil). (The second scorer was unaware of this allocation or this design.) The second scorer was asked to identify whether the given response was an acceptable response for the target. For a response to score a point it must have been deemed acceptable in the target condition and unacceptable in the foil condition. [A response was deemed acceptable where the communication partner had gathered some relevant, useful information from the message, that was close to the target and which would be useful as a means of conveying the message. For example: **Target:** Chair. **Possible Acceptable Responses**: Sitting; sofa Acceptable because sitting and sofa relate to 'chair' and convey useful information related to the item chair. **Target:** Table. **Possible Unacceptable Responses**: Wall; toothbrush - Not acceptable because wall and toothbrush do not relate closely to 'table' and do not convey information closely related to the item ‘table’]

An example of the score award system is provided in supplementary data table 1 (below).

**Supplementary data table 1. Example of ambiguous responses judged by a second scorer and the corresponding system for awarding points**

| **ITEM NUMBER** | **EXAMPLE RESPONSE** | **TARGET (List 1)** | **Judged acceptable** | **FOIL (List 2)** | **Judged acceptable?** | **Score Awarded** |
| --- | --- | --- | --- | --- | --- | --- |
| 1 | Sleeping | Bed | YES | Spider | NO | 1 |
| 2 | Diving | Swimming | YES | Football | YES | 0 |
| 3 | Newspaper | Piano | NO | Book | YES | 0 |

***Rationale for scoring process***

The decision to extend scoring from a 0 or 1 judgment of incorrect/correct to a 0, 1, 2 measure for degree of accuracy was taken to account for the clinical observation that although participants may not always be able to accurately convey the precise target when gesturing, their actions often convey related semantic meaning to the communication partner. This additional information may give rise to more accurate funneling of information to support both parties in moving towards the intended shared understanding. Progress towards this improved mutual understanding achieved within this fashion is not captured within a scoring system that accounts for target or synonymous answers alone (i.e. a 0 or 1 bimodal response). For this reason, the decision to include a further level of scoring as a measure of communicative success was taken. Items judged acceptable within this framework were awarded one point and items which matched precisely with the target or a synonym, were awarded two points. This difference in weighting is intended to reflect the level of communicative benefit gained by reaching this response – with an exact match being more beneficial than an acceptable (but less precise) alternative.

**
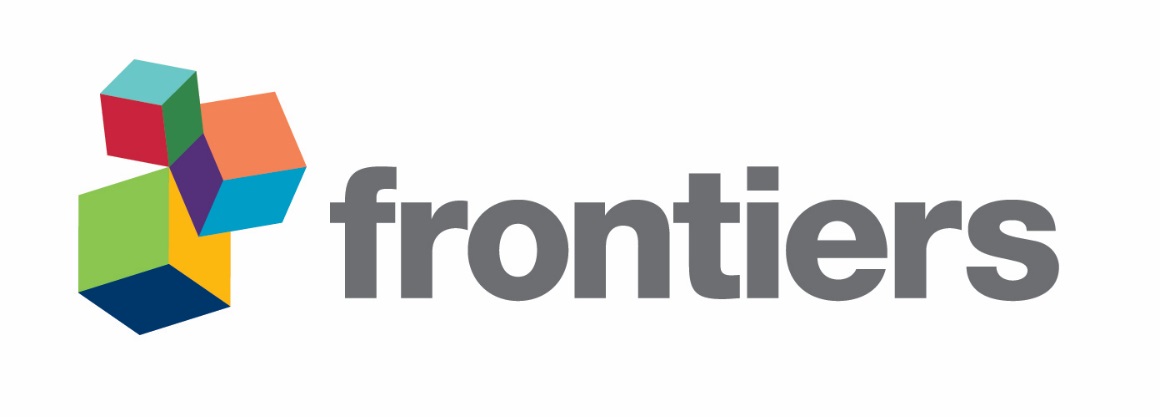
**
